# Supplementary material for: BOLD Coupling between Lesioned and Healthy Brain Is Associated with Glioma Patients’ Recovery
Source: Cancers (Basel). 2021 Oct 6;13(19):5008. doi: 10.3390/cancers13195008 (PMC8508466; doi:10.3390/cancers13195008)
Supplement: Supplementary file 1 [file cancers-13-05008-s001.zip › cancers-1329042-supplementary.pdf]

cancers

MDPI

Supplementary Materials

BOLD Coupling between Lesioned and Healthy Brain Is Associated with Glioma Patients' Recovery

Rafael Romero-Garcia, Michael G. Hart, Richard A. I. Bethlehem, Ayan Mandal, Moataz Assem, Benedicto Crespo-Facorro, Juan Manuel Gorriz, Gladstone Austin Amos Burke, Stephen J. Price, Thomas Santarius, Yaara Erez and John Suckling

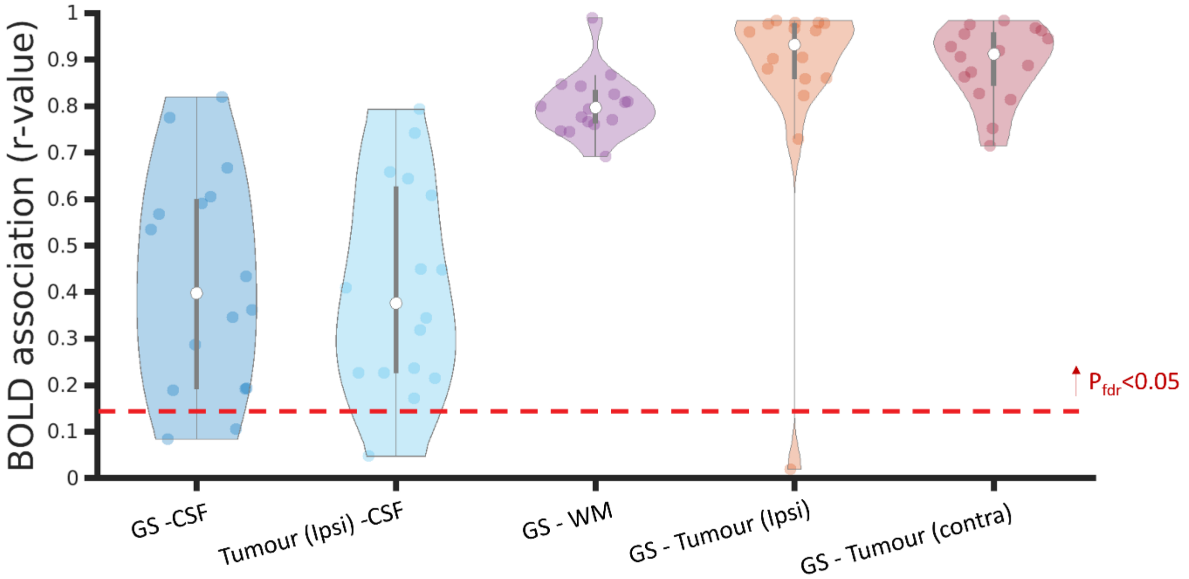

Figure S1. Correlation distribution across brain tumour patients (represented by individual dots) between BOLD signals derived from different tissue compartments: Gray Matter (that is, from which the GS is extracted), Cerebrospinal Fluid (CSF), White Matter (WM), Tumour (Tumour -ipsi-) and cortical regions contralateral to the tumour (Tumour -contra-). R-values over the dashed red line represent significant associations.

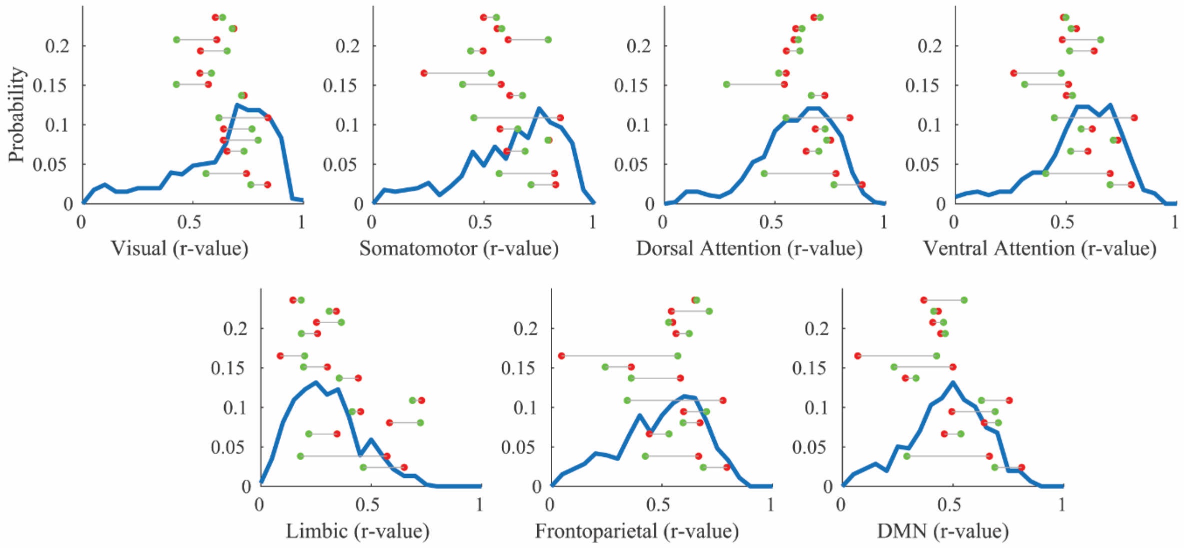

Figure S2. Average correlation within canonical networks in HC and brain tumour patients before and after surgery. Blue lines represent the distribution of the average correlation within each of the 7 canonical networks for 653 HCs. Individual

points illustrate the within network correlation for each brain tumour patient in the hemisphere containing the tumour before (red points), and immediately after surgery (green points). The Y-axis represents probability values only for HC, not for the individual points.

Table S1. Demographic and pathological information. # Number of patient; M, Male; F, Female; L, Left; R, Right; SFG, Superior Frontal Gyrus; MFG, Middle Frontal Gyrus; IFG, Inferior Frontal Gyrus; ITG, Inferior Temporal Gyrus; MTG, Middle Temporal Gyrus; SMA, Supplementary Motor Area; RT, radiotherapy; Astro – astrocytoma; GBM – glioblastoma multiforme; Oligo – oligodendroglioma; Lesion Vol Pre/Post – Total volume occupied by the tumour (Pre) and total amount of damaged tissue (Post) according to the lesion mask manually drawn on the MPRAGE image and refined with Unified Segmentation Lesion toolbox.

**#**	**Age**	**Gender**	**Hand-edness**	**Presen-tation**	**Hemi**	**Location**	**Histology (WHO grade)**	**Molecular signature**	**Tumour/ Lesion Vol (Pre/Post) (cm^3)**	**Treat-ment**
**1**	41	F	L	Seizures	L	Frontal	Oligo (II)	*IDH* Mutated *1p19q* Lost *ATRX* Retained	166.8 94.5	Chemo-RT
**2**	32	M	R	Seizures	R	Insula	Astro (II)	*IDH* WT *1p19q* - *ATRX* Lost	83.2 47.8	Chemo-RT
**3**	26	M	R	Seizures	L	Temporal / Insula	GBM (IV)	*IDH* Mutated *1p19q* - *ATRX* Lost	59.0 30.1	Chemo-RT
**4**	49	F	R	Incidental	R	Insula	Oligo (II)	*IDH* Mutated *1p19q* Lost *ATRX* Retained	31.5 37.5	Observatio n
**5**	55	F	R	Recu-rrence	L	Frontal / SFG / frontal pole	Oligo (II)	*IDH* Mutated *1p19q* Lost *ATRX* Retained	21.1 43.4	Observatio n
**6**	22	F	L	Seizures	R	Frontal / IFG	Ganglio-glioma (I)	*IDH* - *1p19q* - *ATRX* -	4.4 7.5	Observatio n
**7**	29	M	R	Seizures	R	Frontal / SFG & MFG	Astro (III)	*IDH* Mutated *1p19q* Negative *ATRX* Lost	31.8 70.0	Chemo-RT
**8**	29	M	R	Seizures	R	Frontal / MFG	Astro (III)	*IDH* Mutated *1p19q* Negative *ATRX* Lost	22.6 34.2	Chemo-RT
**9**	50	M	L	Seizures	L	Temporal / ITG	GBM (IV)	*IDH* WT *1p19q* Negative *ATRX* Retained	15.6 15.7	Chemo-RT
**10**	38	F	R	Seizures	L	Frontal / MFG	Oligo (II)	*IDH* Mutated *1p19q* Lost *ATRX* Retained	34.7 54.9	Chemo-RT
**11**	29	M	R	Seizures	L	Frontal / SFG / frontal pole	Astro (II)	*IDH* Mutated *1p19q* Negative *ATRX* Lost	52.1 116.9	Observatio n
**12**	33	F	R	Head-aches	L	Temporal / MTG	Astro (III)	*IDH* Mutated *1p19q* Negative *ATRX* Lost	87,0 84,8	Chemo-RT
**13**	27	F	R	Seizures	L	Superior Temporal Gyrus	Ganglioglio ma (I)	*IDH* WT *1p19q* - *ATRX* -	7.6 8.5	Observatio n
**14**	56	F	R	Seizures	L	Superior Temporal Gyrus	Astro (II)	*IDH* Mutated *1p19q* Negative *ATRX* Lost	37.4 19.2	Chemo-RT

**15**	32	M	R	Seizures	L	Superior Temporal Gyrus	Astro (III)	*IDH* Mutated<br>*1p19q* -<br>*ATRX* Lost	18.0<br>30.3	Chemo-RT
**16**	27	M	R	Seizures	L	SFG/SMA & Pre-central	GBM (IV)	*IDH* WT<br>*1p19q* Negative<br>*ATRX* Retained	73.6<br>43.7	Chemo-RT
**17**	30	M	R	Seizures	L	Inferior frontal	Astro (III)	*IDH* Mutated<br>*1p19q* -<br>*ATRX* Lost	26.2<br>17.4	Chemo-RT

Table S2. MRI scans and neuropsychological assessments completed by each participant. # Number of patient.

	MRI scanning				*Neuropsychological assessment*	
#	**Pre-operative**	**Post-operative**	**Month 3**	**Month 12**	**Pre-operative**	**Follow-up**
**1**	Completed	Completed	Completed	Completed	Completed	Completed
**2**	Completed	Not completed	Completed	Completed	Completed	Not completed
**3**	Completed	Completed	Not completed	Not completed	Completed	Not completed
**4**	Completed	Completed	Completed	Not completed	Completed	Completed
**5**	Completed	Completed	Completed	Completed	Completed	Completed
**6**	Completed	Completed	Completed	Completed	Completed	Completed
**7**	Completed	Completed	Not completed	Completed	Completed	Completed
**8**	Completed	Completed	Completed	Completed	Completed	Completed
**9**	Completed	Completed	Not completed	Not completed	Completed	Not completed
**10**	Completed	Completed	Completed	Completed	Completed	Completed
**11**	Completed	Completed	Not completed	Not completed	Completed	Completed
**12**	Completed	Completed	Completed	Completed	Completed	Completed
**13**	Completed	Completed	Completed	Not completed	Completed	Completed
**14**	Completed	Completed	Completed	Not completed	Completed	Completed
**15**	Completed	Completed	Completed	Completed	Completed	Completed
**16**	Completed	Completed	Completed	Not completed	Completed	Completed
**17**	Completed	Completed	Completed	Completed	Completed	Completed
